# Angiomotin prevents pluripotent lineage differentiation in mouse embryos via Hippo pathway-dependent and -independent mechanisms

**DOI:** 10.1038/ncomms3251

**Published:** 2013-08-01

**Authors:** Chuen Yan Leung, Magdalena Zernicka-Goetz

**Affiliations:** 1 The Wellcome Trust/Cancer Research UK Gurdon Institute, the Henry Wellcome Building of Cancer and Developmental Biology, University of Cambridge, Tennis Court Road, Cambridge CB2 1QN, UK; 2 Department of Physiology, Development and Neuroscience, University of Cambridge, Downing Street, Cambridge CB2 3EG, UK

## Abstract

Cell identity is specified in the early mammalian embryo by the generation of precursors for two cell lineages: the pluripotent inner cell mass and differentiating trophectoderm. Here we identify Angiomotin as a key regulator of this process. We show that the loss of Angiomotin, together with Angiomotin-like 2, leads to differentiation of inner cell mass cells and compromised peri-implantation development. We show that Angiomotin regulates localization of Yap, and Yap-binding motifs are required for full activity of Angiomotin. Importantly, we also show that Angiomotin function can compensate for the absence of Lats1/2 kinases, indicating the ability of Angiomotin to bypass the classical Hippo pathway for Yap regulation. In polarized outside cells, Angiomotin localizes apically, pointing to the importance of cell polarity in regulating Yap to promote differentiation. We propose that both Hippo pathway-dependent and Hippo pathway-independent mechanisms regulate Yap localization to set apart pluripotent and differentiated lineages in the pre-implantation mouse embryo.

## Abstract (web summary)

Angiomotins retain the transcription co-activator YAP in the cytoplasm and thereby regulate the Hippo pathway in mammalian cultured cells. Here Leung and Zernicka-Goetz show that Angiomotin family members prevent the differentiation of inner cell mass cells in the mouse blastocyst, via both Hippo pathway-dependent and -independent mechanisms.

## Graphical abstract

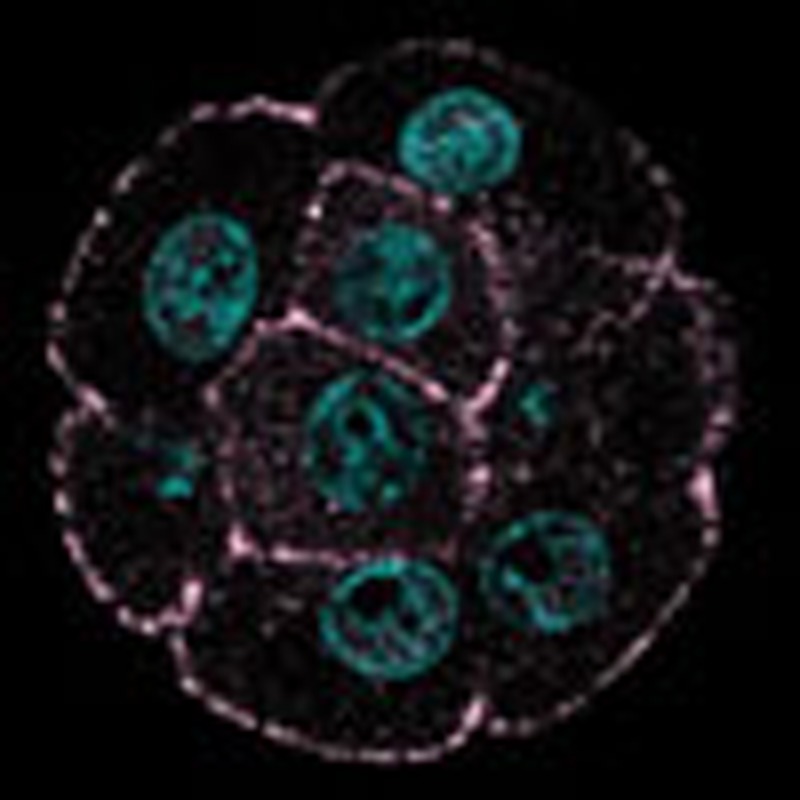

As development progresses, cell identity must be correctly specified. In early mammalian development, three cell types have to be set apart by the blastocyst stage: epiblast, which will give rise to the embryo proper, trophectoderm (TE) and primitive endoderm (PE), which will provide the extra-embryonic structures such as the placenta and yolk sac. Specifying these three cell lineages is mediated through three waves of asymmetric cell divisions that start at the eight-cell stage and generate nonpolar inside and polar outside cells^1^,^2^. Inside cells constitute the so-called inner cell mass (ICM) and will develop further as either the pluripotent epiblast or the PE, largely depending on whether an inside cell is generated by the first, second or third wave of asymmetric division^3^,^4^. Outside cells will progressively differentiate into TE. Thus, inside and outside cells are progenitors for pluripotent ICM and TE lineages, respectively.

Three lines of evidence indicate that regulation of expression of the transcription factor Cdx2 is particularly crucial to initiate diversification of ICM and TE lineages^5^,^6^,^7^,^8^. First, Cdx2 is the first transcription factor identified thus far whose expression becomes restricted to the outside cells as soon as they form^6^, whereas pluripotency factors are initially expressed in both inside and outside cells. Second, Cdx2 downregulation leads to the upregulation of pluripotency genes in outside cells and, consequently, inhibition of TE formation and developmental arrest^5^,^6^. Finally, experimental upregulation of Cdx2 in inside cells prevents pluripotency of the ICM^8^.

The differential expression of Cdx2 between ICM and TE progenitor cells is brought about along two major routes. We recently found that one of these routes involves asymmetric, apical localization of *Cdx2* mRNA that results in outside cells inheriting more *Cdx2* mRNA than inside cells upon asymmetric cell division^6^,^8^. Accordingly, preventing the asymmetric localization and inheritance of *Cdx2* mRNA leads to the accumulation of Cdx2 protein in inside cells and, consequently, inhibition of pluripotency genes such as *Nanog*^8^. In a second route, transcriptional control is achieved through localization of a transcriptional co-activator Yap, which permits Tead4-mediated transcription of *Cdx2* (ref. 99). In the polarized outside cells, Yap is nuclear, whereas in inside non-polarized cells Yap is cytoplasmic. This localization of Yap in mouse embryos is surprising because in other model systems Yap is retained in the cytoplasm of polarized cells, through the activity of apically associated proteins such as Crumbs, Merlin, Expanded and Angiomotin (Amot)^10^,^11^,^12^,^13^. This raises the question of whether, and if so how, cell polarity might be involved in regulation of Yap localization in the mouse embryo. To resolve this conundrum, it has been proposed that the differential localization of Yap in the mouse is achieved through differential Hippo signalling due to differences in the cell–cell contacts between the inside and outside cells^9^. However, whether there might be an alternative mechanism involved in controlling Yap localization to regulate expression of differentiation genes such as *Cdx2* and lineage segregation in mouse development has remained unknown.

Here we wished to elucidate further the mechanisms underlying the regulation of *Cdx2* expression and the potential role of cell polarity in affecting localization of Yap to control this process. To this end, we chose to follow the role of Amot, a protein known to bind to both tight-junction proteins and also to Yap/Taz^11^,^12^,^13^,^14^,^15^,^16^,^17^. Amot is known to have an important role in a variety of developmental process^18^,^19^, but any role for Amot in the pre-implantation embryo has been unknown. Our results indicate that cell polarity contributes to directing the localization of Amot and of Yap in early mouse development. Importantly, Yap localization and therefore Cdx2 expression is regulated not only through a Hippo pathway-dependent mechanism but also through a Hippo pathway-independent mechanism.

## Results

### Differential expression of Amot in ICM and TE precursors

**Figure 1:**
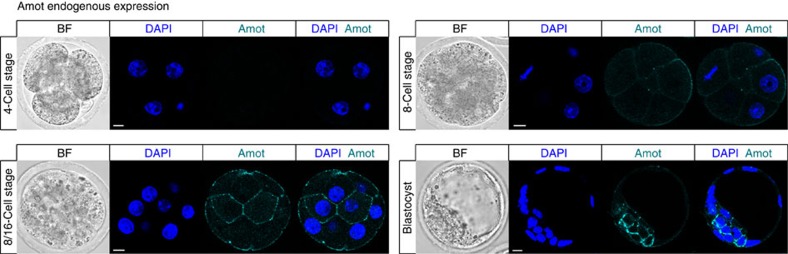
Amot expression and localization in pre-implantation embryos. Immunofluorescent confocal images of Amot. Amot is first detected at the compacted eight-cell stage, where it is localized to the membrane (N=16). At the 8- to 16-cell transition, Amot is localized apically in the outside cells and throughout the membrane of the inside cells (*N*=12). By the blastocyst stage, Amot is much more abundant in the ICM compared with the TE (*N*=18). Weak Amot staining can be seen on the apical domain of TE cells. Yellow areas and dotted lines mark inside cells. Scale bar, 10 μm.

To determine the potential function of Amot in regulating cell-lineage specification in mouse embryos, we first examined its expression throughout pre-implantation development. Interrogation of our previously generated genome-wide microarray data set^20^ indicated that *Amot* expression rises from the four-cell stage to peak at the early blastocyst stage. This prompted us to analyse the localization pattern of Amot protein, using an antibody that detects the C-terminal part of both p130 and p80 isoforms (Fig. 1). We were able to detect Amot protein from the late eight-cell stage onwards when it becomes localized to the membrane, consistent with Amot being a membrane-associated protein^21^. However, when progenitors of the ICM and TE lineages become set apart, at the 8- to 16-cell transition, Amot becomes localized throughout the membrane in the inside cells, whereas in outside cells it is restricted to the apical domain (Fig. 1). As development progresses, Amot becomes highly expressed in the ICM but barely detectable in the TE (Fig. 1). These results indicate that Amot becomes expressed and localized differentially between the progenitors of the pluripotent ICM and the differentiating TE lineages.

### Amot is required to prevent differentiation of the ICM

**Figure 2:**
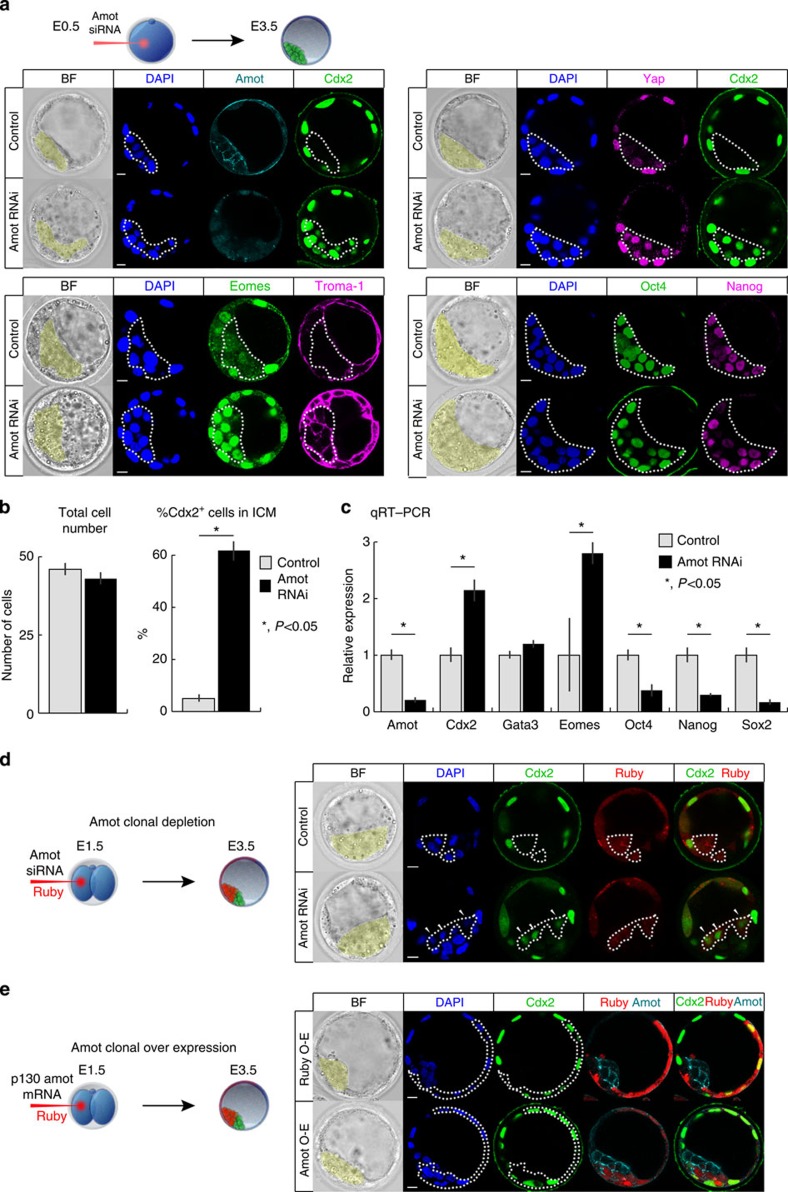
Amot prevents differentiation of the ICM. (**a**) Immunofluorescent confocal images of TE/ICM marker genes of control and *Amot*-RNAi blastocysts. Control (*N*=20)/*Amot* siRNA (*N*=30) were injected into zygotes and cultured to blastocysts; Amot protein was undetectable in *Amot*-RNAi blastocysts. In the absence of Amot, TE markers Cdx2 (*N*=30), Eomes (*N*=16) and Ck-8 (antibody: Troma-1) (*N*=16) become upregulated in the ICM and Yap localized to the nuclei of ICM cells (*N*=12) in contrast to control blastocysts, where Yap is cytoplasmic (*N*=12). Pluripotency markers Oct4 and Nanog (*N*=11) are still visible. Yellow areas and dotted lines mark inside cells. Scale bar, 10 μm. (**b**) Total cell number of *Amot*-RNAi versus control blastocysts. The total number of cells between *Amot*-RNAi (46.3±2cells, *N*=20 embryos) and control blastocysts (43.2±2cells, *N*=30 embryos) is not significantly different (*P*=0.51, Student’s *t*-test). The percentage of Cdx2-positive cells in the ICM of *Amot*-RNAi blastocysts (61.8±5%, *N*=434 cells, 30 embryos) is greater than in control blastocysts (5.0±2%, *N*=311 cells, 20 embryos). Error bars represent s.e.m.. Student’s *t*-test was used to test significance. (**c**) qRT–PCR of whole embryos, comparing expression levels of several TE/ICM markers of *Amot*-RNAi (*N*=60) versus control (*N*=60) blastocysts. Error bars represent s.e.m. Student’s *t*-test was used to test significance. (**d**) Immunofluorescence of embryos clonally depleted of Amot, showing that Amot governs cell fate in a cell-autonomous manner. One blastomere of two-cell embryos was injected with control/*Amot* siRNA and Ruby mRNA, and cultured to blastocysts. Ruby-positive cells mark the injected clone. In the ICM of the mosaic *Amot*-RNAi embryos, only the Ruby-positive cells expressed Cdx2 (*N*=21 cells) (arrows). Yellow areas mark the ICM. Dotted lines mark Ruby-positive ICM cells. Scale bar, 10 μm. (**e**) Immunofluorescence of embryos that are clonally overexpressing Amot, showing that Amot is not sufficient to drive cells to ICM. One blastomere of two-cell embryos was injected with Ruby mRNA only or Amot and Ruby mRNA, and cultured to blastocysts (*N*=10). Ruby-positive cells mark the overexpressing clone. Yellow areas mark the ICM. Dotted lines mark Ruby-positive cells. Scale bar, 10 μm.

To determine the role of Amot, we used RNA interference (RNAi) to specifically downregulate its expression from the zygote stage^22^. *Amot* small interfering RNAs (siRNAs) were injected into the zygote, together with Ruby mRNA as a marker of successful injection, and developmental progression was compared with embryos injected with control siRNA (Fig. 2a). Three different siRNAs for *Amot* were injected and the siRNA resulting in the highest knockdown efficiency (79.1±4% by qRT–PCR) (Fig. 2c) was used in subsequent experiments. We also confirmed Amot depletion at the protein level (Fig. 2a). *Amot* depletion did not prevent blastocyst formation (Fig. 2a) and did not affect total cell number (Fig. 2b). However, we found that Cdx2, critical for TE differentiation, was detectable not only in the TE but also within the ICM in *Amot*-RNAi embryos (Fig. 2a). Overall, 61.8±5% of total ICM cells in *Amot-*RNAi blastocysts were clearly expressing Cdx2, as compared with 5.0±2% in control embryos (Fig. 2b). As we observed that the absence of Amot leads to Cdx2 expression in the ICM, we examined the localization of the co-activator of *Cdx2* expression, Yap^9^. We found Yap to be localized to the nuclei of ICM cells upon *Amot*-RNAi (Fig. 2a). Amot depletion also led to the expression of other TE-marker genes such as *Eomes* and *Ck-8* (Troma-1) in the ICM (Fig. 2a). The use of an siRNA control, together with rescue experiments (described later), indicate that the *Amot*-RNAi phenotype is specific and not an artefact of either microinjection or off-target effects.

We next wished to determine the consequences of *Amot*-RNAi on pluripotency factors. qRT–PCR of *Amot*-RNAi blastocysts revealed that the transcript levels of *Oct4, Nanog* and *Sox2* were significantly reduced to 38.5±11%, 29.8±2% and 17.4±5%, respectively (*P*<0.05, Student’s *t*-test) (Fig. 2c), although Oct4 and Nanog proteins were still detectable (Fig. 2a). In contrast, and in agreement with our earlier analyses, the transcript levels of *Cdx2* and *Eomes* increased to 214.9±18% and 281.0±19% of control levels, respectively, whereas *Gata3*, another TE-marker gene, was not significantly elevated (Fig. 2c). Together, these results suggest that, in the absence of Amot, although cells can be allocated to the ICM, they express TE-specific genes and the pluripotency network (*Oct4*, *Nanog* and *Sox2*) becomes suppressed.

### Amot acts in a cell-autonomous manner

As Amot localizes to membranes, we wondered whether it might be able to signal and affect the fate of neighbouring cells. To investigate this, we injected *Amot* siRNA into one blastomere at the late two-cell embryo, co-injecting Ruby mRNA as a lineage marker, and analysed embryo development at the blastocyst stage. We found that the Amot-depleted clones contributed to both inside (53.3±1%) and outside (46.7±1%) cells, suggesting that the cell allocation process *per se* was unaffected. In all embryos, only the clones of Amot-depleted cells were expressing Cdx2 in the ICM (Fig. 2d), suggesting that loss of Amot in one cell does not affect a neighbouring cell, and therefore Amot regulates cell fate in a cell-autonomous manner.

### Amot overexpression is insufficient to drive cells to ICM

After establishing that Amot is necessary for correct ICM specification, we sought to determine whether Amot is sufficient to induce pluripotent ICM fate when overexpressed. To examine this, we injected p130-*Amot* mRNA into one blastomere at the late two-cell embryo, co-injecting Ruby mRNA as a marker (Fig. 2e). We confirmed Amot overexpression by immunofluorescence (Supplementary Fig. S1). Analysis of cell allocation revealed that the Amot overexpressing clone contributed to both ICM (46.8±2%) and TE (53.2±2%) lineages, suggesting that Amot is insufficient to drive cells into the ICM. Interestingly, by the blastocyst stage, Amot remained highly expressed only in the ICM and markedly downregulated in the TE (Supplementary Fig. S2), pointing to a potential regulatory mechanism in the TE that degrades Amot to prevent incorrect fate specification.

### Depletion of Amot with Amotl2 enhances ICM differentiation

**Figure 3:**
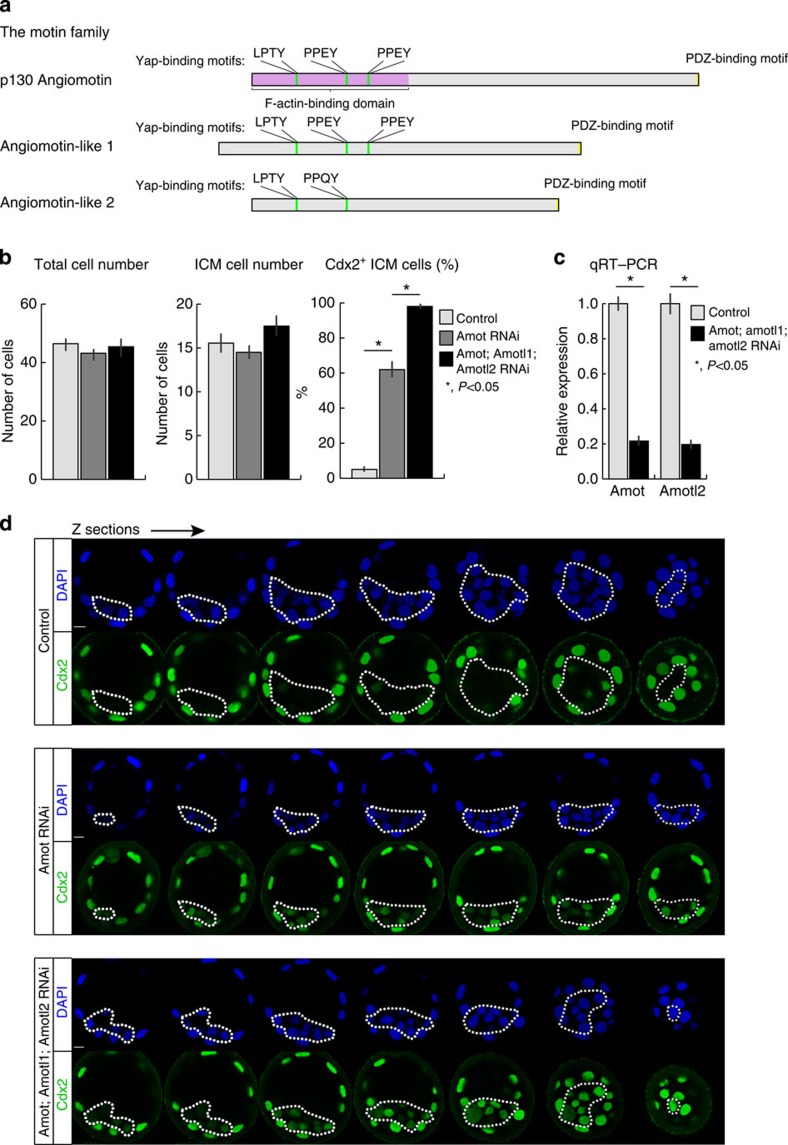
Co-depletion of motin family members enhance ICM differentiation. (**a**) The motin family proteins with key regions outlined. (**b**) Total cell number and ICM cell number are not significantly different between control (*N*=21), *Amot*-RNAi (*N*=31) and *Amot*; *Amotl1*; *Amotl2*-RNAi (*N*=12) blastocysts. However, in the triple motin-RNAi embryos, 98.0±1% of ICM cells of the triple RNAi embryos were Cdx2 positive at the blastocyst stage (*N*=210 cells, 12 embryos) compared with the 61.8±5% of *Amot*-RNAi (*N*=434 cells, 30 embryos) and 5.0±2% of control embryos (*N*=311 cells, 21 embryos). Error bars represent s.e.m. Student’s *t*-test was used to test significance. (**c**) qRT–PCR of whole embryos, comparing expression levels of *Amot* and *Amotl2* in control embryos (*N*=21) and triple knockdown embryos (*N*=21). *Amot* and *Amotl2* were downregulated to 21.6±3% and 19.6±3%, respectively, relative to control levels. *Amotl1* expression could not be detected. Error bars represent s.e.m. Student’s *t*-test was used to test significance. (**d**) Z-section series of immunofluorescent confocal images of control, *Amot*-RNAi (*N*=30) and *Amot*; *Amotl1*; *Amotl2*-RNAi (*N*=12) blastocysts, with the highest proportion of Cdx2-expressing cells in the triple knockdown. Dotted lines outline the ICM. Scale bar,: 10 μm.

Our results indicate that the lack of Amot leads to the expression of differentiation genes within the ICM and downregulation of pluripotency. It has been reported that *Amot*^*−/−*^ embryos can progress beyond implantation^18^,^19^, prompting us to test whether Amot-related proteins might be able to compensate for Amot function. Amot belongs to the motin family that contains two other members: Amot-like 1 (Amotl1) and Amot-like 2 (Amotl2) (Fig. 3a)^23^, both of which have overlapping functions with Amot^11^,^12^,^13^,^24^. To eliminate all three motins, we injected *Amot, Amotl1, Amotl2* siRNAs into zygotes and examined developmental progression. We confirmed knockdown by qRT–PCR: *Amot* transcripts were depleted to 21.6±3% and *Amotl2* to 19.6±3%, whereas *Amotl1* transcripts were undetectable (Fig. 3c). We found that co-depletion of *Amot* and *Amotl2* permitted blastocyst formation (9/10, 90%; control siRNA: 9/10, 90%) and did not affect the total cell number or number of ICM cells per embryo (Fig. 3b). Strikingly, however, the proportion of Cdx2-expressing cells within the ICM was much higher in the combined motin-RNAi embryos than upon depletion of *Amot* alone. Nearly all (98.0±1%) ICM cells of the *Amot-* and *Amotl2*-RNAi embryos were Cdx2 positive compared with the 61.8±5% of *Amot* only-RNAi and 5.0±2% of control embryos (Fig. 3b,d). This suggests that Amot and Amotl2 both function to suppress *Cdx2* expression in the ICM.

### Amot depletion compromises developmental potential

**Figure 4:**
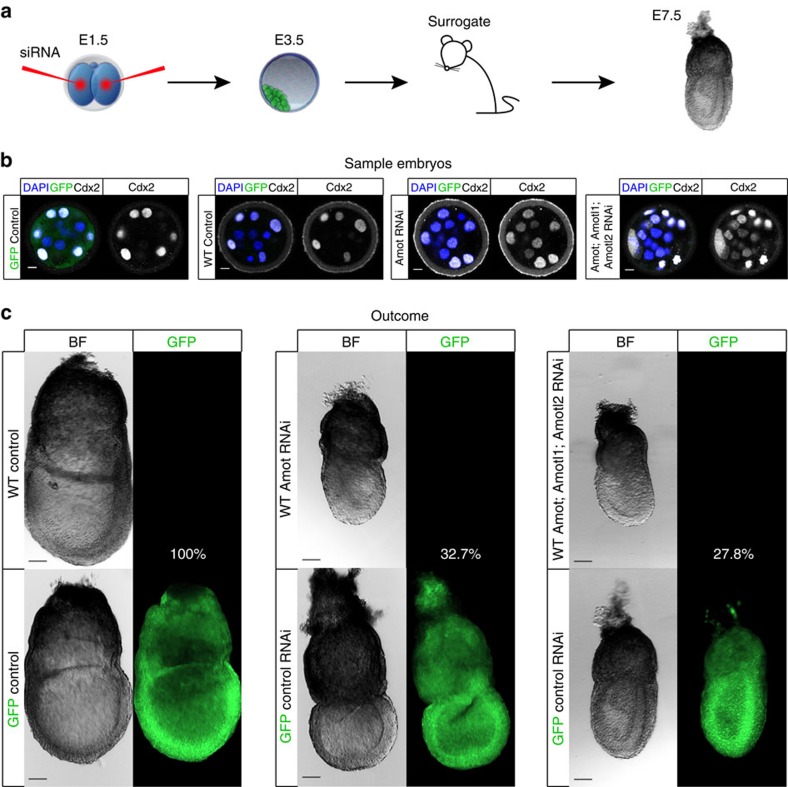
Loss of AMOT compromises the developmental potential of peri-implantation embryos. (**a**) Experimental outline: siRNA was injected to both blastomeres of two-cell-stage embryo, cultured to E3.5 and transferred to 2.5 dpc pseudo-pregnant recipients. The embryos were recovered 5 days later at E7.5. Embryos were injected with either control siRNA, *Amot* siRNA or a combination of *Amot*, *Amotl1* and *Amotl2* siRNA. For each of the three groups (control, *Amot*-RNAi and *Amot*; *Amotl1*; *Amotl2*-RNAi), 20 WT embryos and 20 GFP embryos were transferred to two surrogates. (**b**) Embryos from each group were sampled to confirm their phenotype following RNAi. *Amot*-RNAi embryos (*N*=9) and *Amot*; *Amotl1*; *Amotl2*-RNAi (*N*=6) expressed CDX2 in the inside cells, whereas in GFP (*N*=10) and WT (*N*=10) embryos injected with control siRNA CDX2 expression was restricted to the outside cells. (**c**) The outcome of the embryo transfers. To assess for developmental success, the embryos were recovered at E7.5. For the control-RNAi group, out of the 40 embryos transferred, 38 embryos were recovered, of those 20 (100%) were GFP+ and 18 (90%) were GFP−. For *Amot*-RNAi, out of 40 embryos transferred, 22 embryos were recovered, of those 17 (85%) were GFP+ and 5 (25%) were GFP−. For the *Amot*; *Amotl1*; *Amotl2*-RNAi, out of 40 embryos transferred, 20 embryos were recovered, of those 16 (80%) were GFP+ and 4 (20%) were GFP−. After normalizing for the transfer efficiency of the different recipients and background of the embryos, 100% of the control-RNAi embryos progressed successfully to E7.5, compared with 32.7% of Amot-RNAi embryos (*P*<0.05, *χ*^2^-test, compared with control) and 27.8% of Amot;Amotl1;Amotl2-RNAi embryos (*P*<0.05, *χ*^2^-test, compared with control). There was no significant difference between the developmental success of *Amot*-RNAi and *Amot*; *Amotl1*; *Amotl2*-RNAi embryos (*P*=0.67, *χ*^2^-test). Scale bar, 100 μm.

To determine whether the ICM differentiation caused by the loss of Amot would affect development *in vivo*, *Amot*-RNAi and *Amot*, *Amotl1, Amotl2*-RNAi embryos were transferred into pseudo-pregnant surrogates to assess their developmental potential (Supplementary Table S1). siRNAs were injected into both blastomeres at the two-cell stage, rather than the zygote, to prolong the RNAi effect (Fig. 4a). GFP transgenic embryos injected with control siRNA were used as transfer controls to normalize for transfer efficiency in different recipients. After injection, the embryos were cultured to the blastocyst stage and sampled to confirm their respective phenotypes (Fig. 4b). To assess for successful development beyond implantation, the embryos were recovered at E7.5 (Fig. 4c). After normalizing for the transfer efficiency of the different recipients and background of the embryos, we found that 100% of the control-RNAi embryos progressed successfully to E7.5, compared with 32.7% of *Amot-*RNAi embryos (*P*<0.05, *χ*^2^-test, compared with control) and 27.8% of the combined motin-RNAi embryos (*P*<0.05, *χ*^2^-test, compared with control). There was no significant difference between the developmental success of *Amot*-RNAi and combined motin-RNAi embryos (*P*=0.67, *χ*^2^-test), although loss of all three motins might lead to a more severe outcome (Supplementary Table S2). Overall, our result suggests that loss of Amot compromises development, potentially as a result of a more differentiated ICM, signifying an important role for Amot in early mouse development.

### p130-Amot is the isoform involved in cell fate regulation

**Figure 5:**
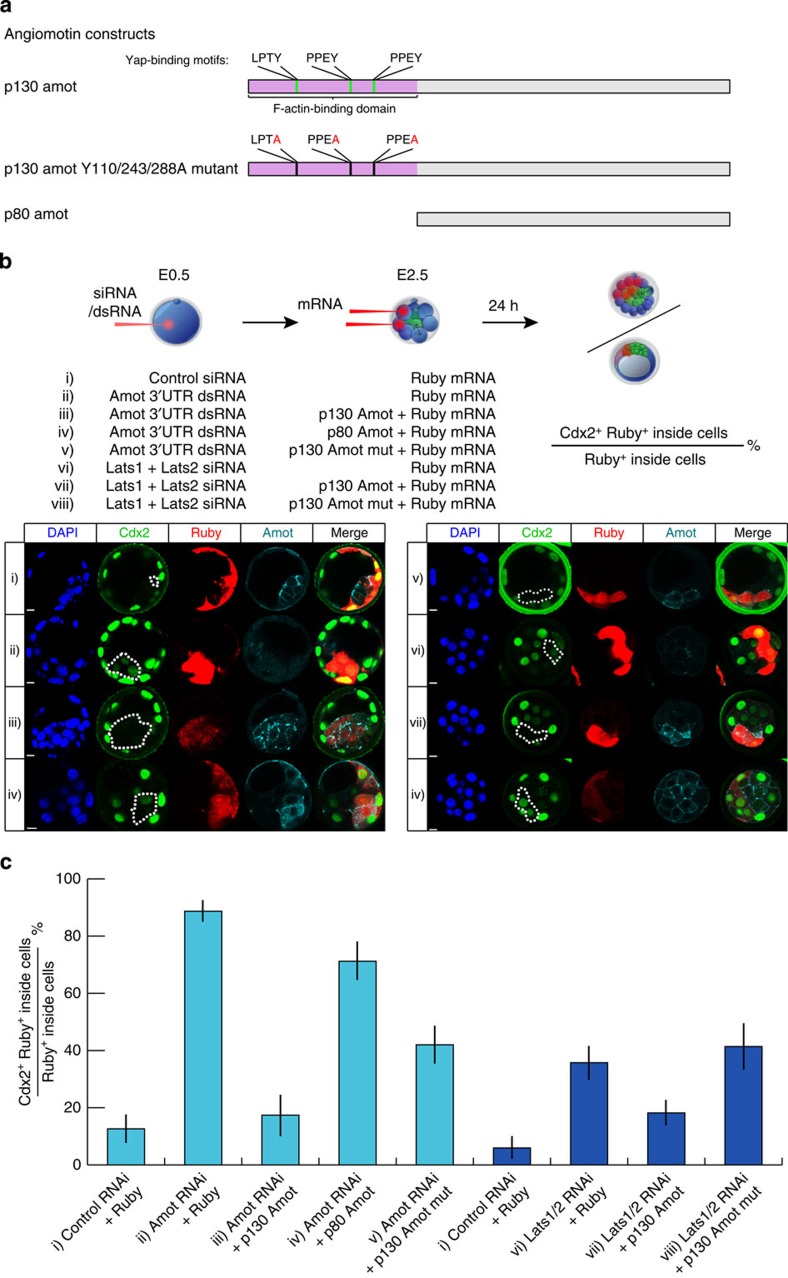
p130-Amot functions independently of the Hippo pathway kinases Lats1/2. (**a**) Amot constructs with key regions outlined. The p80- and p130-Amot isoforms are identical, except the p130 isoform has an N-terminal extension, which contains three Yap-binding motifs. (**b**) Eight sets of experiments (i)–(viii) are shown, which follow the same experimental outline: siRNA/dsRNA was injected into the zygote and mRNA was injected into two or three blastomeres at the eight-cell stage. Cdx2-positive inside cells from all eight sets of experiments were counted manually. The phenotype was quantified by calculating the percentage of Cdx2-positive cells from the Ruby-positive inside cells at the blastocyst stage (or the morula stage in the case of *Lats1/2* knockdown). Panels show confocal images of representative embryos from each set of experiments. Dotted lines mark Ruby-positive inside cells. Scale bar, 10 μm. (**c**) Combined quantifications from the experiments outlined in (**b**). Number of biological replicates: (i) *N*=41 cells, 15 embryos; (ii) *N*=96 cells 34 embryos; (iii) *N*=53 cells, 35 embryos; (iv) *N*=44 cells, 11 embryos; (v) *N*=20 cells, 40 embryos; (i) *N*=22 cells, 9 embryos; (vi) 139 cells, 24 embryos; (vii) *N*=145 cells, 43 embryos; (viii) *N*=58 cells, 18 embryos. Error bars represent s.e.m. This quantification shows that the p130, but not the p80, isoform is able to rescue the *Amot* phenotype and the Yap-binding motifs are required for the full activity of p130-Amot. *Lats1/2* depletion leads to an increased Cdx2-positive inside cell number, but overexpression of p130-*Amot* is able to prevent *Cdx2* expression in the absence of Lats1/2, demonstrating a Hippo pathway-independent mechanism. Confirming this conclusion overexpressing the *Amot* mutant for Yap-binding motifs in a *Lats1/2*-RNAi background does not affect the *Lats1/2* phenotype.

The Amot protein is expressed as two isoforms, p80 and p130 (refs 21, 25). The p130 isoform has a unique N-terminal extension and the rest of the protein is identical to the p80 isoform (Fig. 5a). To determine which isoform is required for the first cell lineage specification, we depleted *Amot* with a dsRNA targeting the 3′-UTR in the zygote and then attempted to rescue *Amot*-RNAi effect with either the p80- or p130-Amot isoforms at the eight-cell stage. Successful knockdown by the dsRNA was confirmed by qRT–PCR (Supplementary Fig. S3). For the rescue, we co-injected either p80- or p130-*Amot* mRNA, with Ruby mRNA as a marker, into 2–3 blastomeres at the eight-cell stage. To quantify the extent of the rescue, we determined the percentage of the Ruby- and Cdx2-positive cells in the ICM (Fig. 5b). In control blastocysts, 12.6±5% of the Ruby-marked ICM cells were Cdx2-positive (Fig. 5c) (most likely owing to inherited Cdx2 protein from an outside mother cell). This value increased significantly (*P*<0.05, Student’s *t*-test) to 88.7±4% of the *Amot*-RNAi/Ruby-expressing embryos (Fig. 5c). When we injected p130-*Amot* mRNA to the *Amot*-RNAi embryos, the proportion of Cdx2-expressing cells in the ICM dropped significantly to 17.4±7%. This value was significantly different (*P*<0.05, Student’s *t*-test) from *Amot*-RNAi/Ruby-expressing embryos and not significantly different from control embryos (*P*=0.84, Student’s *t*-test), indicating that p130-Amot is able to fully rescue the effects of Amot depletion. We found that providing p80-*Amot* into *Amot-*RNAi embryos still resulted in 71.2±7% of ICM cells expressing Cdx2, which was significantly different from the control embryos (*P*<0.05, Student’s *t*-test) (Fig. 5b,c). Therefore, the *Amot*-RNAi effect can be rescued only by the p130 isoform, indicating that p130-Amot function is required to suppress the differentiation pathway in the ICM.

### Yap-binding motifs are involved in Amot function

The N-terminal extension of the p130-Amot contains two PPxY motifs. Mutating the tyrosine residues to alanine in the PPEY motifs abolishes the Yap-binding ability of Amot and its ability to tether Yap in the cytoplasm^11^,^12^,^13^. A third motif conserved across all motins, LPTY, has been suggested to mediate interaction between Yap and Amotl1 (ref. 26). These motifs therefore represent a Yap-dependent but Hippo pathway-independent activity of Amot. We sought to determine whether Amot might function in the mouse embryo independently of the Hippo pathway. We hypothesized that if overexpression of the triple motif mutant (Y110/243/288 A) could rescue the *Amot*-depletion phenotype, Yap-binding motifs are consequently not important. If the opposite is true, it would follow that Amot functions through a Hippo pathway-independent, Yap-binding mechanism. To test this, we depleted Amot, from the zygote as before, and subsequently injected mRNA for Y110/243/288A *Amot* with Ruby mRNA as a lineage marker, into 2–3 blastomeres at the eight-cell stage. We found that, at the blastocyst stage, 42.0±7% of the clonal Ruby-positive ICM cells were expressing Cdx2 (Fig. 5c), which was significantly lower than that in *Amot*-RNAi embryos (88.7±4%, *P*<0.05). This suggests the Yap-binding motifs are required for the full activity of Amot, and therefore Amot function is, in part, Yap binding dependent. In agreement with this, Yap staining in Amot overexpressing embryos suggests possible co-localization between Yap and Amot (Supplementary Fig. S4), as previously shown in MCF10A cells^11^. In some embryos (Fig. 5c, panel v), however, Cdx2 was not detectable in the ICM upon the Y110/243/288A mutant overexpression, suggesting that the mutant Amot was partially able to suppress Cdx2 expression. Indeed, the proportion of Ruby- and Cdx2-positive ICM cells following injection of mRNA for Y110/243/288A *Amot* was 42.0±7%, an intermediate value between 17.4±7% of the control and 88.7±4% of the *Amot*-RNAi embryos (Fig. 5c). These results indicate that the Yap-binding sites are required for the full activity of Amot, raising the possibility that pluripotent lineage specification can be regulated in a Yap-binding-dependent but a Hippo pathway-independent manner.

### Amot compensates for Lats1/2 function

The above results opened up a possibility that Amot can regulate Yap localization, and consequently Cdx2 expression, independently of the Hippo pathway. If this is the case, Amot overexpression should suppress Cdx2 expression in the absence of Hippo pathway kinases, Lats1/2, which phosphorylate Yap to prevent its nuclear localization^9^. To directly test this, we eliminated *Lats1* and *Lats2* transcripts by injecting zygotes with *Lats1/2* siRNAs and, at the eight-cell stage, injected 2–3 blastomeres with either Ruby mRNA only or p130-*Amot* mRNA with Ruby mRNA. We verified successful depletion of *Lats1/2* by qRT–PCR (Supplementary Fig. S5). On assessing at the morula stage, we found that *Lats1/2*-RNAi/Ruby-expressing embryos had 35.7±6% of Ruby-positive inside cells expressing Cdx2, which was significantly more than the 5.9±4% in control-RNAi/Ruby-expressing embryos of the same stage (*P*<0.05, Student’s *t*-test), in agreement with previous report^9^. Importantly, overexpression of *Amot* following Lats1/2 depletion significantly decreased the proportion of Cdx2- and Ruby-positive inside cells to 18.2±4% (Fig. 5c), compared with *Lats1/2-*RNAi/Ruby-expressing embryos (*P*<0.05, Student’s *t*-test). This value was not significantly different from the control-RNAi/Ruby-expressing embryos (*P*=0.06, Student’s *t*-test). These results suggest that by increasing the amount of Amot its Hippo pathway-independent activity can compensate for the loss of the Hippo pathway kinase Lats1/2.

To confirm this conclusion, we next overexpressed Y110/243/288A *Amot* that cannot bind Yap in the *Lats1/2*-RNAi background. To this end, we depleted *Lats1/2* as above, but now co-injected Y110/243/288A Amot, with Ruby mRNA as a lineage tracer, to 2–3 blastomeres at the eight-cell stage. In these embryos, 41.4±8% of the Ruby-positive inside cells were Cdx2 positive, a value not significantly different from the 35.7±6% of the *Lats1/2*-RNAi/Ruby-expressing embryos (*P*=0.58, Student’s *t*-test) (Fig. 5c). Thus, the Y110/243/288A mutant is not able to compensate for the loss of Lats1/2, further pointing to a direct Amot/Yap interaction that bypasses the classical Hippo pathway. On a side note, this also indicates that the *Lats1/2* phenotype is Yap dependent, as only Amot with intact Yap-binding sites can compensate for Lats1/2 function. Taken together, these results further indicate that Amot can function in a Yap-dependent but Hippo pathway-independent manner to specify cell fate in the mouse embryo.

## Discussion

**Figure 6:**
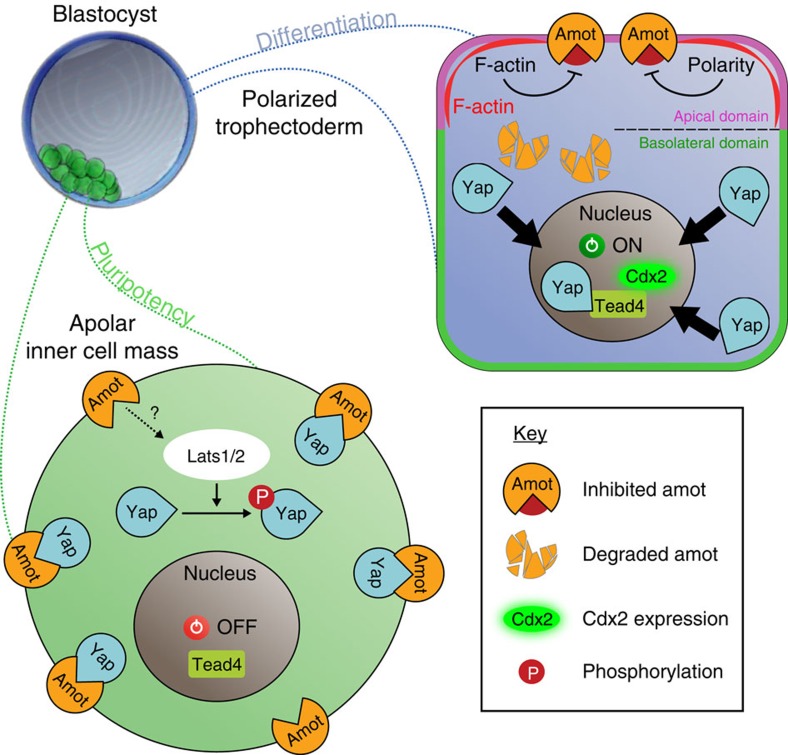
Hippo pathway-dependent and -independent mechanisms in lineage segregation. The results we present here show that Amot becomes enriched in the ICM as cell lineages become progressively established, and that Amot functions to prevent differentiation of the pluripotent lineage. Our results suggest that Amot acts through two routes to prevent ICM differentiation, of which one is dependent and another is independent of the Hippo pathway. The Hippo pathway-independent (Lats1/2–independent) mechanism is likely to involve Amot’s ability to tether Yap to the cytoplasm/membrane, as we find that Yap-binding motifs of Amot are essential for its full activity. But, it can also involve Amot interacting with actin/cell polarity molecules. The Hippo pathway-dependent mechanism is likely to involve Amot’s role in activating Lats1/2 (as shown in^24^) to allow phosphorylation of Yap, which prevents its transition to the nucleus^9^. In agreement, we find that mutation of the Yap-binding sites does not completely abolish the activity of Amot. Combined, this results in Yap being sequestered from the nucleus and therefore the expression of TE-genes, such as Cdx2, is switched off and the ICM pluripotency maintained. In contrast, in TE cells, most of Amot is degraded and the remaining Amot is sequestered to the apical domain. We hypothesize that in this location Amot is shielded from binding Yap through its interactions with F-actin and/or polarity factors. In agreement with this hypothesis, Amot is reported to associate with actin^32^ and tight-junction proteins via its PDZ-binding domain in tissue culture cells^14^,^15^,^16^,^17^. This suggests that cell polarity is involved in allowing Yap to translocate to the nucleus to act, together with Tead4, to stimulate *Cdx2* expression and TE differentiation.

Analyses of spatial and temporal expression patterns together with the downregulation and overexpression studies we present here identify Amot as a new important regulator of the first cell fate specification in the mouse embryo. We find that Amot becomes enriched in the ICM versus TE progenitor cells as development progresses; Amot is critical to prevent expression of TE-marker genes in the pluripotent lineage; ICM differentiation is significantly enhanced by co-depletion of Amot with Amotl2; Amot regulates localization of Yap; full activity of Amot is dependent on the Yap-binding motifs, suggesting a Hippo pathway-independent function; Amot function can compensate for the absence of Lats1/2, supporting its ability to function independently of the Hippo pathway; and mutating the Yap-binding motifs affects, but does not completely abolish, Amot activity, suggesting that Amot might also act through an alternative mechanism, such as through activating Lats1/2 via the Hippo pathway or independent of Yap and, finally, that Amot depletion, together with depletion of Amotl2, results in compromised development at peri-implantation stages. Overall, our results identify a role of Amot in the first-lineage specification to prevent an inappropriate cell fate program in the pluripotent ICM and suggest that Amot has two distinct roles in this process, sequestering Yap, which is Hippo pathway independent, and also activating the Hippo pathway kinase Lats1/2 (Fig. 6).

Our results indicate that loss of Amot results in the upregulation of genes that promote differentiation and, consequently, downregulation of pluripotency genes such as *Oct4*, *Nanog* and *Sox2* in the ICM. Despite this, we observe that some pluripotency-associated transcription factors are still present, possibly as a result of pre-existing protein. This finding is likely to explain why Amot^−/−^ embryos can survive through implantation and arrest only at early post-implantation stages^18^,^19^. This could also be due to developmental plasticity, as the mouse embryo has been shown to require establishment of only four pluripotent cells by implantation to support its development to birth^4^. Finally, genetic redundancy could also be a contributing factor, as we show here that simultaneous depletion of Amot and Amotl2 lead to a much stronger phenotype than depletion of Amot on its own.

As development progresses, Amot becomes upregulated in ICM but not TE progenitors. A deep sequencing analysis of global gene expression patterns between ICM and TE progenitors after the first wave of asymmetric division, at the 16-cell stage, reveals similar levels of *Amot* mRNA (Krzysztof Wicher, Sarah Graham and Magdalena Zernicka-Goetz, unpublished observations). Although this does not rule out differential transcription later, our results suggest that Amot enrichment in the ICM might result from differential stability of the protein between ICM and TE cells. In support of this, we show that when Amot is overexpressed, it is downregulated in TE but not in ICM cells (Supplementary Fig. S2). Moreover, one of the E3 ubiquitin ligases able to promote Amot degradation^27^, NEDD4L, is expressed two-fold higher in TE than in ICM progenitors (Krzysztof Wicher, Sarah Graham and Magdalena Zernicka-Goetz, unpublished observations), thus giving the potential of reducing Amot levels in TE. In addition, cytoplasmic Yap has been found to protect Amotl1 from NEDD4-mediated degradation^26^, raising an interesting possibility that Yap could act in a positive feedback loop to prevent Amot degradation in the ICM.

Two models are often considered for the first cell fate decision in the mouse embryo. The polarity model^1^ stresses the importance of cell polarity in directing cell fate, and the positional model^28^,^29^ stresses the importance of cell position. It has been demonstrated that cell polarity and cell position re-enforce each other^30^, and these two models can be brought together conceptually^31^. Identifying Amot as a cell fate regulator is a molecular step towards this unification, because it lends support to both: Amot becomes enriched in inside but not outside cells (supporting a positional ‘inside-outside’ model) and its localization is polarized in outside cells (supporting a ‘polarity’ model). Apically localized Amot in polarized outside cells may interact with polarity factors, and, in support of this idea, it has been already shown to associate with tight-junction proteins via its PDZ-binding domain^14^,^15^,^16^,^17^. Therefore, we would like to suggest that, in the mouse embryo, as in other model systems, cell polarity is involved in Yap regulation as it can, for example, inhibit Amot’s ability to bind Yap to restrain its translocation to the nucleus (Fig. 6).

The ability of Amot to function through Yap is strongly suggested by the three putative Yap-binding motifs of p130-Amot that are absent in p80-Amot and which we show here are essential for the full activity of Amot. Indeed, Yap seemingly co-localizes with p130-Amot when p130-Amot is overexpressed (Supplementary Fig. S4). Another way to test Amot’s Yap dependence would be through an *Amot*; *Yap* double knockdown. However, interpreting such an experiment would be difficult because of the redundancy of Yap with its homologue Taz^9^ (Supplementary Fig. S6).

We find that Amot is partly functional following mutation of its Yap-binding sites, which suggests that two possible mechanisms of Amot function are tenable. One would reflect the direct interaction between Amot and Yap, independent of the Hippo pathway. The second would reflect the residual function of this Yap-binding defective Amot mutant that would still be able to activate Lats1/2, leading to Yap phosphorylation and cytoplasmic retention (as demonstrated in HEK293 cells^24^). Our results cannot also rule out the possibility that the residual function of this Amot mutant reflects function that is entirely independent of Yap.

The key regulatory domain of Amot appears to be its N-terminal part, as we show that the p80 isoform does not have a role in cell fate regulation. This N-terminal domain could be responsible for activation of Lats1/2-dependent phosphorylation of Yap, as supported by *in vitro* data^24^. We also consider the actin-binding properties of the N-terminal domain of p130-Amot^32^ could relate to its ability to suppress *Cdx2* expression in the pluripotent lineage. In agreement with this hypothesis, it has been reported that an increase in filamentous actin results in increased nuclear Yap and activation of Yap target genes^33^,^34^,^35^,^36^,^37^,^38^. Finally, we show that overexpressing p130-Amot prevents the expression of *Cdx2* in inside cells lacking Lats1/2. Hence, in the absence of the Hippo pathway, high levels of Amot are sufficient to sequester most of the Yap molecules from the nucleus. Taken together, our results suggest that both Hippo pathway-dependent and -independent processes are involved in regulating Yap localization to generate pluripotent cells in the mouse embryo.

## Methods

### Embryo collection and culture

Animals were maintained in the Animal Facility of Gurdon Institute at 12:12 light cycle and provided with food and water *ad libitum*. All experiments were conducted in compliance with Home Office regulations. For embryo collection, 4- to 6-week-old F_1_ generation mice from the C57Bl6 and CBA strains were superovulated with 10 IU of pregnant mare serum gonadotropin (PMSG; Intervet) and 10 IU human chorionic gonadotropin (hCG; Intervet) 48 h later and mated with F1 (C57Bl6 × CBA) male mice. Embryos were dissected out of the oviducts into M2 medium. For zygote recovery, cumulus cells were removed by hyaluronidase treatment (1 mg ml^−1^ in M2). Embryos were cultured in drops of KSOM media (Millipore) under paraffin oil in 5% CO_2_ at 37 °C.

### Immunohistochemistry and analysis

Embryos were fixed in 4% PFA for 20 min and washed twice for 5 min in PBS-T (PBS with 0.1% Tween). They were then permeabilized with 0.5% Triton X-100 in PBS for 20 min and washed three times for 5 min in PBS-T. The embryos were incubated with primary antibodies diluted in 3% BSA (Sigma) for 1 h. Cdx2 antibody (Biogenex) was used at a dilution of 1:200; Oct4 antibody (Santa Cruz) at 1:200; Nanog antibody (2BScientific) at 1:200; Yap antibody (Santa Cruz) at 1:400; Eomes antibody (Abcam) at 1:250; Troma-1 at 1:100; and Amot antibody (Santa Cruz) at 1:2,000. The embryos were then washed three times for 5 min in PBS-T. Secondary antibodies (Invitrogen, AlexaFluor) and DAPI (Invitrogen) were applied for 1 h at a dilution of 1:400 in 3% BSA (Sigma). After washing three times for 5 min in PBS-T, the embryos were washed overnight in M2 medium. Embryos were imaged on glass-bottom dishes rather than on slides in order to avoid flattening the embryo. Confocal images were captured with a Leica SP5 confocal microscope with a Z-resolution of 4 μm. All images are layer-normalized using IMARIS to account for signal intensity differences from different sections. To count total cell numbers, DAPI was used as a nuclear marker to represent each cell. Acquisition of Cdx2 stainings for cell counting was performed using identical settings on a Leica SP5. To count Cdx2-positive cells, the confocal z-stacks were first exported to IMARIS and layer-normalized using the built-in function. The normalized sections were then assessed for the presence of Cdx2 signal. Any nuclei with signal detectable by eye were scored as ‘Cdx2 positive’. Cells were then manually counted in ImageJ using the MTrackJ plug-in. Student’s *t*-test, unless otherwise stated, was used to test statistical significance.

### RNAi reagents and construct preparation

p130- and p80-*Amot* were cloned from mouse kidney cDNA and sequence verified. mRNA was synthesized using the mMESSAGE mMACHINE Kit (Life Technologies). dsRNA was designed and evaluated using E-RNAi, a web application for the design of RNAi reagents^39^. The DNA template of the dsRNA was amplified from mouse kidney cDNA, and dsRNA was synthesized from DNA using the T7 MEGAscript kit (Life Technologies). The dsRNA was annealed by heating to 70 °C for 5 min and allowed to cool at room temperature for 2 h. It was treated with RNAse T1/A and Proteinase K to remove ssRNA and proteins. Site-directed mutagenesis of the PPxY motifs was performed via overlap extension PCR. Four PCR fragments were amplified from *Amot* DNA using mutagenic primers, with two fragments overlapping over each PPxY motif. All four fragments were pooled, annealed and PCR amplified into one complete piece of DNA, which was sequence verified. The following is the sequence of *Amot* siRNA used: 5′-CAGGAGAAGCCTACTCAGCTA-3′. Primers used for cloning are as follows: p80-*Amot* 5′-TTTTTGGATCCACCATGCCTCGGGCTCAG-3′ 5′-TTCGTTTGCGGCCGCTTAGATGAGATATTCC-3′; p130-*Amot* 5′- TTTTTTTTTTGGATCCACCATGAGAAGTTCTGACGATCAGCC-3′ 5′-TTTTTTTTTTGCGGCCGCTTAGATGAGATATTCCACC-3′. The following primers were used to generate the p130-Amot Y110/243/288A mutant: 5′-TTTTTTTTTTGGATCCACCATGAGAAGTTCTGACGATCAGCC-3′; 5′-GACCTTGGCCTCTTCGGCGGTTGGGAGTTCTTC-3′; 5′-GAAGAACTCCCAACCGCCGAAGAGGCCAAGGTC-3′; 5′-TTTTTTTTTTATGCATCCTTCTAATCTCGCCCTCCAGCTTGTTCC-3′; 5′-CCCTTGAAAGGGGCTTCTGGCGGGGGACCTC-3′; 5′-GAGGTCCCCCGCCAGAAGCCCCTTTCAAGGG-3′; 5′-CTGCCCCGGCCTCAGGGGGGTGCTGATA-3′; 5′-TATCAGCACCCCCCTGAGGCCGGGGCAG-3′. The following primers were used to generate dsRNA targeting the 3′-UTR of *Amot*: 5′-TAATACGACTCACTATAGGGTGTGTTTGGGGAGAAAAGGA-3′; 5′-TAATACGACTCACTATAGGGAAGTCCAGGAAAAGGCCTGA-3′.

### Embryo microinjection

Microinjection was performed on a Leica DM IRB with attached Leica micromanipulators. Injections were administered via air pressure using an Eppendorf Femtojet microinjector, and negative capacitance was used to facilitate membrane penetration. All injections were carried out on embryos suspended in M2 medium under paraffin oil on a depression slide. Successful microinjections were confirmed by the presence of fluorescent markers co-injected with the constructs. Ruby mRNA (0.4 mg ml^−1^) was co-injected for all experiments except in the rescue experiments where 0.4 mg ml^−1^ Gap-RFP was co-injected to mark the membrane without interfering with the cytoplasmic Ruby introduced at the eight-cell stage.

### qRT–PCR

RNA was extracted from embryos using the Arcturus PicoPure RNA Isolation Kit. qRT–PCR was carried out using SYBR Green in a StepOne Plus Real-time PCR machine (Applied Biosystems). *Gapdh* was used as an endogenous control. The following primers were used: *Gapdh* 5′-AGAGACGGCCGCATCTTC-3′ 5′-CCCAATACGGCCAAATCCGT-3′; *Amot* 5′-GATGTGCAACCCAGATAAGCC-3′ 5′-TCTCTGCATCAGGCTCTTGC-3′; *Cdx2* 5′-AAACCTGTGCGAGTGGATG-3′ 5′-TCTGTGTACACCACCCGGTA-3′; *Eomes* 5′-CGGCCTACCAAAACACGGAT-3′ 5′-AAGCCGTGTACATGGAATCGTA-3′; *Gata3* 5′-CCGAAACCGGAAGATGTCTA-3′ 5′-AGATGTGGCTCAGGGATGAC-3′; *Oct4* 5′-TTGGGCTAGAGAAGGATGTGGTT-3′ 5′-GGAAAAGGGACTGAGTAGAGTGTGG-3′; *Nanog* 5′-GGTTGAAGACTAGCAATGGTTCTGA-3′ 5′-TGCAATGGATGCTGGGATACTC-3′; *Sox2* 5′-TGCTGCCTCTTTAAGACTAGGG-3′ 5′-CGCCGCGATTGTTGTGATT-3′; *Lats1* 5′-AACAAGTTCTTCCCGGAGCC-3′ 5′-ACCATGTCCTCATCAAAGCCA-3′; *Lats2* 5′-GAATGCGGGATGTGACCAG-3′ 5′-TACTCCAAGGCAGCTTCGAT-3′.

## Author contributions

All experiments were performed in M.Z.G.’s laboratory by C.Y.L. The data were analysed and interpreted by C.Y.L. and M.Z.G. The manuscript was written by C.Y.L. and M.Z.G.

## Additional information

**How to cite this article:** Leung, C.Y. and Zernicka-Goetz M. Angiomotin prevents pluripotent lineage differentiation in mouse embryos via Hippo pathway-dependent and independent mechanisms. *Nat. Commun.* 4:2251 doi: 10.1038/ncomms3251 (2013).

## Supplementary Material

Supplementary Information Supplementary Figures S1-S6 and Supplementary Tables S1-S2
