# Supplementary material for: Angiomotin prevents pluripotent lineage differentiation in mouse embryos via Hippo pathway-dependent and -independent mechanisms
Source: Nat Commun. 2013 Aug 1;4:2251. doi: 10.1038/ncomms3251 (PMC3741640; doi:10.1038/ncomms3251)
Supplement: Supplementary Information — Supplementary Figures S1-S6 and Supplementary Tables S1-S2 [file ncomms3251-s1.pdf]

SUPPLEMENTARY INFORMATION

Angiomotin prevents pluripotent lineage differentiation in mouse embryos via Hippo pathway-dependent and independent mechanisms

Chuen Yan Leung and Magdalena Zernicka-Goetz

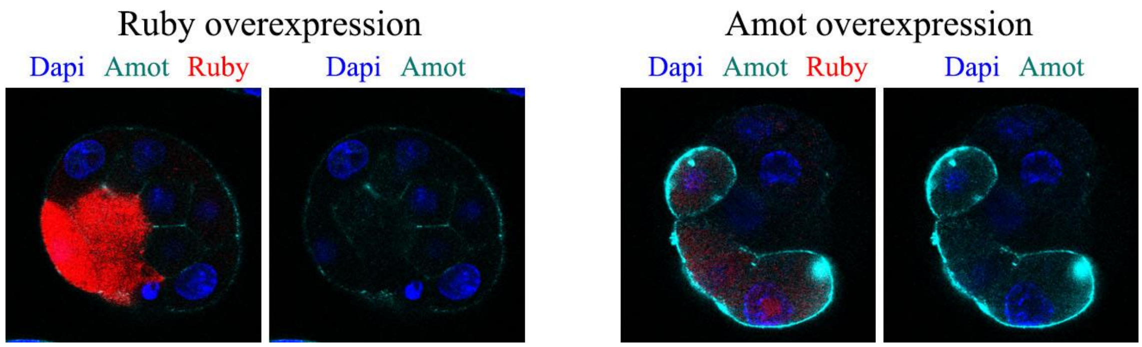

Supplementary Figure S1: Validation of Amot over-expressing construct

One 2-cell blastomere was injected with either 1.0µg/µl Ruby mRNA as a control or 0.8µg/µl *Amot* mRNA with 0.2µg/µl Ruby mRNA and cultured for 24 hours (N=9). Protein expression was confirmed with immunofluorescence. Scale bars: 10µm.

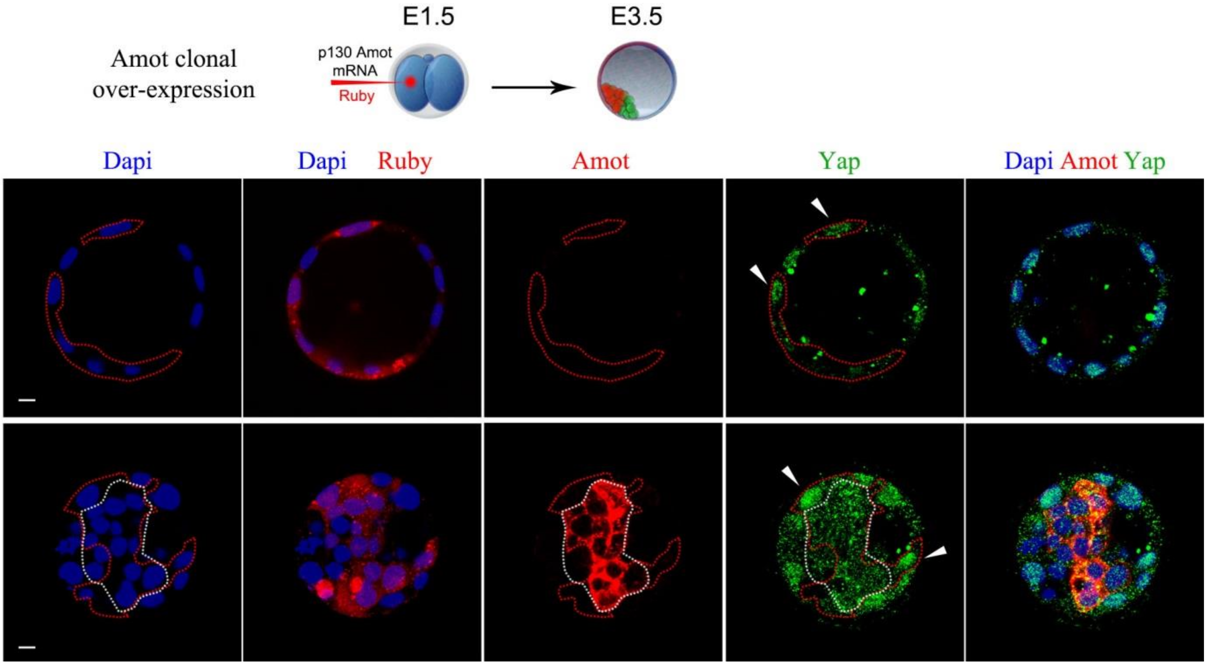

Supplementary Figure S2: Amot is down-regulated in outside cells after 48 hours of Amot over-expression

One blastomere of the 2-cell stage embryo was injected with 0.8µg/µl of p130 *Amot* mRNA and 0.2µg/µl Ruby mRNA to mark the injected clones. Red outlines mark the Ruby-positive cells, white outline mark the inside cells, white arrows point to nuclear Yap accumulation in outside cells of the over-expressing clones. Over 48 hours Amot over-expression became restricted to the inside cells, whereas in the outside cells Amot became dramatically down-regulated (N=16). Scale bars: 10µm.

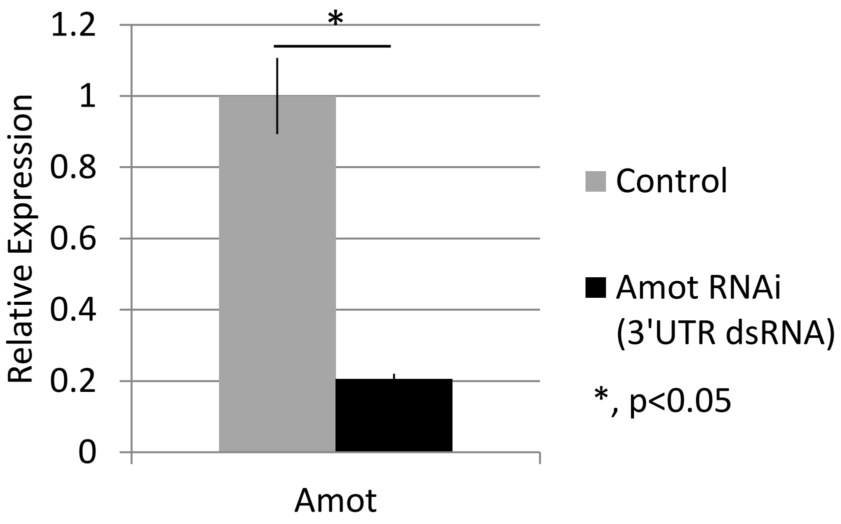

Supplementary Figure S3: qRT-PCR of *Amot*-RNAi embryos with a dsRNA targeting the 3'UTR of *Amot*

Injection of a dsRNA targeting the 3'UTR of *Amot* into zygotes depleted *Amot* transcripts by 79.4±1% when assessed at the blastocyst stage (N=20). Error bars represent standard error of the mean. Student's t-test was used to test significance.

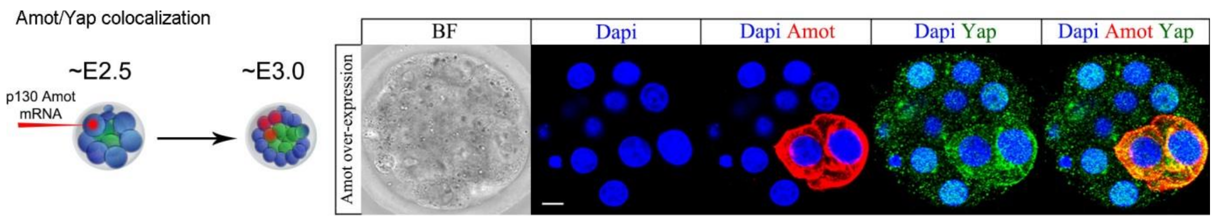

Supplementary Figure S4: Amot over-expression demonstrates Amot/Yap co-localisation.

One 8-cell blastomere was injected with 0.8μg/μl p130 *Amot* mRNA and cultured for 12 hours (N=13). The altered localisation of Yap suggests it co-localises with Amot, indicating a direct interaction between Amot and Yap in the embryo. This direct interaction represents a Hippo pathway-independent mechanism, as Yap is directly sequestered from the nucleus by Amot. Scale bars: 10μm.

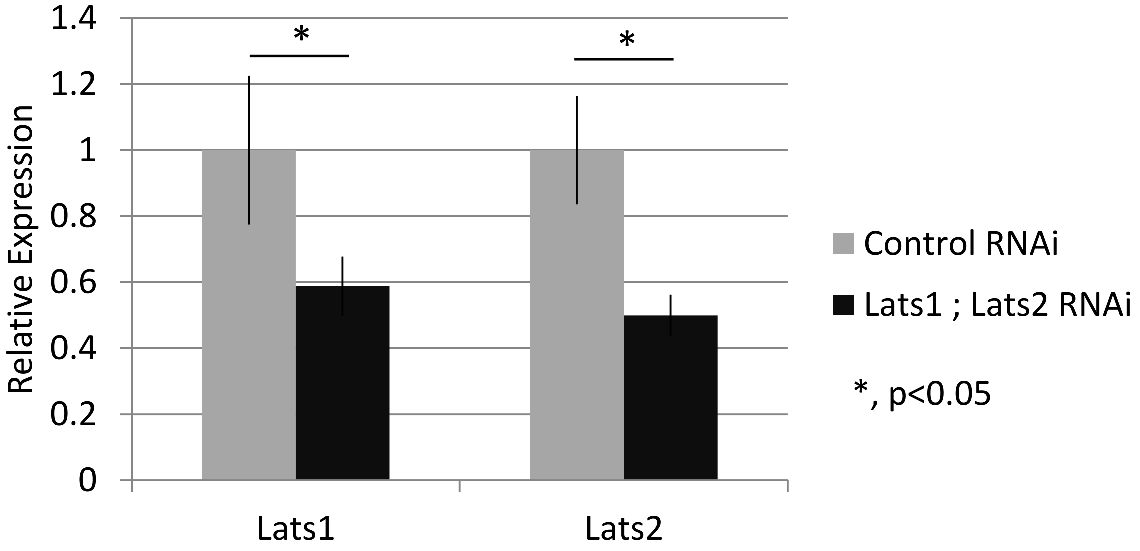

Supplementary Figure S5. qRT-PCR of *Lats1*; *Lats2*-RNAi embryos

Injection of a combination of 3 siRNAs against *Lats1* and 3 siRNAs against *Lats2* into zygotes depleted *Lats1* transcripts by 41±9% and *Lats2* transcripts by 50±6% when assessed at the blastocyst stage (N=18). Error bars represent standard error of the mean. Student's t-test was used to test significance.

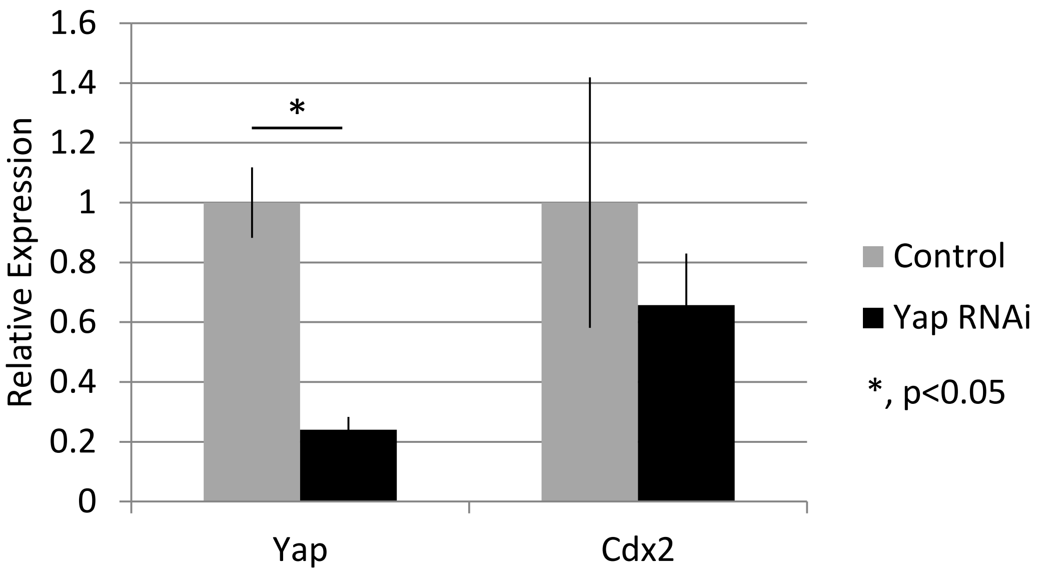

Supplementary Figure S6: qRT-PCR of Yap-RNAi embryos

Injection of a mixture of three different siRNAs targeted against *Yap* into zygotes was able to deplete *Yap* transcripts by 76±4% after 2 days (N=20). However, *Cdx2* transcripts was not significantly reduced (N=20). This is most likely due to functional redundancies between Yap and its homologue Taz. Error bars represent standard error of the mean. Student's t-test was used to test significance.

Supplementary Table S1: Experimental design of the embryo transfer experiment

Genotype	Injection	Embryos transferred	Surrogates
GFP	Control siRNA	20 (Group 1)	2
WT	Control siRNA	20 (Group 1)	
GFP	Control siRNA	20 (Group 2)	2
WT	*Amot* siRNA	20 (Group 2)	
GFP	Control siRNA	20 (Group 3)	2
WT	*Amot; Amotl1; Amotl2* siRNA	20 (Group 3)	

3 groups of embryos were transferred into pseudo-pregnant surrogates. Each group consists of 20 GFP and 20 WT embryos. GFP embryos are always injected with control siRNA. 10 GFP embryos and 10 WT embryos were transferred into one uterine horn of 1 surrogate. In total 2 surrogates were used for each group.

Supplementary Table S2: Outcome of the embryo transfer experiment

<u>**Outcome**</u>	Embryos transferred	Embryos recovered	GFP+ embryos	GFP- embryos	Normalized for background and transfer efficiency
Group 1: Control	40	38	20 (100%)	18 (90%)	100%
Group 2: *Amot*-RNAi	40	22	17 (85%)	5 (25%)	32.7%
Group 3: *Amot; Amotl1; Amotl2*-RNAi	40	20	16 (80%)	4 (20%)	27.8%

The outcome of the embryo transfers. To assess for developmental success the embryos were recovered at E7.5. For the group 1 (control), out of the 40 embryos transferred, 38 embryos were recovered, of those 20 (100%) were GFP+ and 18 (90%) were GFP-. For *Amot*-RNAi, out of 40 embryos transferred, 22 embryos were recovered, of those 17 (85%) were GFP+ and 5 (25%) were GFP-. For the *Amot; Amotl1; Amotl2*-RNAi, out of 40 embryos transferred, 20 embryos were recovered, of those 16 (80%) were GFP+ and 4 (20%) were GFP-. After normalizing for the transfer efficiency of the different recipients and background of the embryos, 100% of the control RNAi embryos progressed successfully to E7.5, compared to 32.7% of *Amot*-RNAi embryos ($p<0.05$, $\chi 2$ test, compared to control) and 27.8% of *Amot; Amotl1; Amotl2*-RNAi embryos ($p<0.05$, $\chi 2$ test, compared to control). There was no significant difference between the developmental success of *Amot*-RNAi and *Amot; Amotl1; Amotl2*-RNAi embryos ($p=0.67$, $\chi 2$ test).
